# Homœopathic Therapeutics in Dental Pathology

**Published:** 1888-12

**Authors:** W. Irving Thayer

**Affiliations:** Brooklyn, N. Y.


### HOMŒOPATHIC THERAPEUTICS IN DENTAL PATHOLOGY.

BY W. IRVING THAYER, D.D.S., M.D., BROOKLYN, N. Y.

Read before the New Jersey State Dental Society, Asbury Park, N.J., July 18, 1888.

Homœopathic therapeutics does not consist in the dilution or size of the dose; but "the healing power of medicine rests upon its faculty of producing symptoms *similar* to the disease and superior to it in strength; so that each individual case of disease is most certainly, fundamentally and rapidly extinguished and cancelled by a drug which is more potent than the disease, and capable of producing in the body symptoms most similar to and completely resembling the totality of those of the disease;" be it by the action of a drachm of the crude drug or by the one-thousandth centesimal trituration.

The nearer one selects a drug whose pathogenesis corresponds to the totality of symptoms found objectively and subjectively in the patient, the higher can he run up his remedy and the quicker will he obtain curative results.

The pathogenesis of a drug is the symptoms that will be noted by a well person who takes the crude drug, or some one of its triturations or dilutions, for a series of hours, days or weeks.

A continuous proving of sugar, chloride of sodium, starch or pepper—all articles of food—will produce certain pathological symptoms. If, then, such simple substances will produce abnormal symptoms, what shall be the result of a continued proving of aconite, arsenic, belladonna, aurum, bryonia, causticum, china, crotion tiglium, eupatorium perfolatum, ferrum, iodium, ipecacuanha, creosote, mercurius corrosivus or bichloride of mercury, nux vomica, phosphorus, platina, sepia, spongia, silicia, sulphur, tellurium, plumbago, thuja and zinc, to say nothing of the many dozens of other remedies?

I am quite well aware, that the rule amongst the profession is, when a practitioner finds a pulp that has long been exposed, to proceed to devitalize that pulp. That this may be the best possible method of procedure, for some pulps will hardly admit of a doubt. But under other conditions I should consider it against the best interest of the patient to destroy that pulp.

Two things are as patent as the noon-day sun; the one is that the Almighty created that pulp for specific purposes; the other is that the patient, by his neglect, has done his best to defeat the purposes of his Creator.

The question now arises: What shall the party of the third part do in the premises? According to the feeble intelligence of the writer, if compelled by this high court to answer the question, he would say, save that pulp alive if possible! Other practitioners of greater experience would advise to destroy the pulp every time. If asked why they would so proceed, their answer would be because, sooner or later, say in a year or two, that pulp will surely die! Yes, indeed! "it is appointed unto man once to die, and after that the judgment."

Should a patient come to me with an exposed pulp that had long given him trouble, with severe congestion and a throbbing and beating pain; he desires to save that tooth; and if the cavity is so arranged that I can get a good view of the pulp, horns and all, I save that pulp alive. I have yet to see a tooth with a live and healthy pulp, with an alveolar abscess on its fang, or tendency thereto. I have yet to see a tooth, black, dried and brittle, that has a live and healthy pulp. These are some of reasons for objecting to destroy a living pulp.

It appears to be a very lame and poor argument for me to say, "I always kill exposed pulps, because sooner or later they will die, and nine times out of ten, one will have a putrescent nerve and alveolar abscess to deal with." This may be the sad experience of some gentlemen; but with me it has been different.

It is not claimed that every pulp can be saved, but the large majority can be.

It is no trouble for one to save a freshly exposed pulp that has been exposed by the burr. The reason for this is that there is no acute or chronic inflammation. Dress with carbolic acid, creosote, or the bichloride of mercury to provide against the microbes; then gently cover the exposed portions with oxyphosphate; gently, without pressure. A freshly exposed pulp will tolerate *gentle* contact, but will always rebel against pressure; but, in cases where there is or where there has been acute or chronic pulpitis, or any inflammation, no pulp that has ever come within the superintendence of your speaker will permit even gentle contact, to say nothing of pressure.

Provisions against contact and pressure are two of the most important matters to be attended to in saving exposed pulps, especially those that have been once inflamed.

It is readily understood that it would be the heigth of folly for one to proceed to cap a nerve while there was any inflammation and without making the parts thoroughly aseptic.

Should I find any inflammation or slumbering irritation, I should, for all acute symptoms, depend on aconite, third centesimal, three drops in a half of a tumbler of water, two teaspoonfuls at a dose once in an hour from two to five or six hours, and then every two to three hours, as I found the case progress. Do not crowd the remedy too fast, or you will produce an aggravation of your symptoms. Always lengthen the time between your doses as the symptoms become less urgent. If one has reason to believe that suppuration is threatening, exhibit mercurius corrosivus in about the third centesimal trituation every three hours for a few times. Mercurius corrosivus is a remedy that has been used for a long time by the homœopathic physicians to prevent suppuration.

I need hardly remind you that our new antiseptic, the bichloride of mercury and mercurius corrosivus, are identically the same drug. The former is used to kill the microbe, and the latter is administered constitutionally to prevent the microbe from forming pus. If one is legitimate, the other, certainly, is reasonable. Thus, again, we find the microbe publishing the nuptial bands, and confirming again that there is one law of cure.

One will not aggravate pulpitis by very light topical applications of a suitable dressing when the pulp is exposed to the air. It is when the original cavity of decay is stopped up tightly that a once inflamed pulp will not permit contact and pressure. It is safe to cap and fill only when there is no pulpitis!

But, sometimes pulpitis supervenes some months after the practitioner has completed his operations, on account of thermal change or other exciting cause. If let alone, and only a palliative treatment is adopted, such as topical application of aconite to the gums and counter irritation with capsicum or iodum, the case will surely go on to suppuration, and the utility of capping exposed nerves be condemned.

In such cases the pain and discomfort arises from an undue accumulation of blood in all the parenchyma of the pulp; and there is pressure upon its nerve filaments and pain. The capillaries have dilated and allowed the blood to engorge itself within their minute canals, and they are incapable of contracting upon their contents, or the bony dentine walls to accommodate this expansion of the whole pulp tissue; and, unless relieved, we shall have arrest of function, death and pus.

Now, what is to be done? Simply to make the arterioles contract upon their contents and expel the static blood. How can this be done? By giving a remedy that will cause just such a condition as we find in the frog's foot, and here we will find that homœopathic therapeutics can have an advantageous position in dental pathology.

It should be remembered that aconite's most useful position is in the primary symptoms, and not after an exudation of lymph. Mercury is the remedy then ; but many times, if too much procrastination has been practiced by the patient, and organized lymph begun to form, nothing but death of the parts and suppuration will be the result.

To resuscitate a drowning man, one must immediately begin artificial respiration, and not wait three or four days to consider the subject. To treat successfully congested pulps, one must begin early.

Those persons who have had exposed nerves capped should be instructed to report early, as soon as the first symptoms show themselves, when, nine times out of ten, a few doses of the third dilution of aconite will completely stop all farther progress of the disease,

A cap must be constructed which is so much concaved that should a little swelling of the pulp in its grief ever occur, it will have room enough to enlarge until you can reduce its size by aconite, belladonna or gelsiminum.

Caps made of beaten silver or gold, no thicker than writing paper, made concave by a punch on a piece of lead or wood by a broken excavator, have served your speaker very satisfactorily. The cap should rest beyond the horns of the pulp, and on solid ground, where no softened dentine can give way and precipitate a pressure.

I will close by calling your attention to one more remedy—the extract of Hamamelis Virginica, for the venous congestion of the gums, after the extraction of the teeth. This extract is highly curative, and will serve a much better purpose than any other mouth-wash with which we are acquainted.

Thus, gentlemen, we have endeavored to prove to you that there is a law of cure, and that homœopathic therapeutics is a valuable aid in dental pathology.

